# C9orf72 poly GA RAN-translated protein plays a key role in amyotrophic lateral sclerosis via aggregation and toxicity

**DOI:** 10.1093/hmg/ddx350

**Published:** 2017-09-13

**Authors:** Youn-Bok Lee, Pranetha Baskaran, Jorge Gomez-Deza, Han-Jou Chen, Agnes L Nishimura, Bradley N Smith, Claire Troakes, Yoshitsugu Adachi, Alan Stepto, Leonard Petrucelli, Jean-Marc Gallo, Frank Hirth, Boris Rogelj, Sarah Guthrie, Christopher E Shaw

**Affiliations:** 1 United Kingdom Dementia Research Institute Centre, Maurice Wohl Clinical Neuroscience Institute, Institute of Psychiatry, Psychology and Neuroscience, King’s College London, Camberwell, London SE5 9NU, UK; 2 Department of Developmental Neurobiology, King’s College London, Guy’s Campus, London SE1 1UL, UK; 3 Department of Neuroscience, Mayo Clinic Florida, Jacksonville, FL 32224, USA; 4 Department of Biotechnology, Jožef Stefan Institute, SI-1000 Ljubljana, Slovenia; 5 School of Life Sciences, University of Sussex, JMS Building, Falmer Campus, Brighton, BN7 9QG UK

## Abstract

An intronic GGGGCC (G4C2) hexanucleotide repeat expansion in*C9orf72* is the most common genetic cause of amyotrophic lateral sclerosis and frontotemporal dementia (C9ALS/FTD). Repeat-associated non-AUG (RAN) translation of G4C2 RNA can result in five different dipeptide repeat proteins (DPR: poly GA, poly GP, poly GR, poly PA, and poly PR), which aggregate into neuronal cytoplasmic and nuclear inclusions in affected patients, however their contribution to disease pathogenesis remains controversial. We show that among the DPR proteins, expression of poly GA in a cell culture model activates programmed cell death and TDP-43 cleavage in a dose-dependent manner. Dual expression of poly GA together with other DPRs revealed that poly GP and poly PA are sequestered by poly GA, whereas poly GR and poly PR are rarely co-localised with poly GA. Dual expression of poly GA and poly PA ameliorated poly GA toxicity by inhibiting poly GA aggregation both *in vitro* and *in vivo* in the chick embryonic spinal cord. Expression of alternative codon-derived DPRs in chick embryonic spinal cord confirmed *in vitro* data, revealing that each of the dipeptides caused toxicity, with poly GA being the most toxic. Further, *in vivo* expression of G4C2 repeats of varying length caused apoptotic cell death, but failed to generate DPRs. Together, these data demonstrate that C9-related toxicity can be mediated by either RNA or DPRs. Moreover, our findings provide evidence that poly GA is a key mediator of cytotoxicity and that cross-talk between DPR proteins likely modifies their pathogenic status in C9ALS/FTD.

## Introduction

Hexanucleotide repeat expansion (G4C2) in the first intron of the *C9orf72* gene is the most common genetic cause of amyotrophic lateral sclerosis (ALS) and frontotemporal dementia (FTD). The number of repeats is polymorphic, with the most common number being two but up to 30 repeats have been detected in healthy controls, whereas disease-affected individuals have many more repeats, ranging from 70 to several thousand (1,2). Three pathomechanisms have been proposed. Firstly, reduced C9orf72 protein levels may inhibit endosomal trafficking, affecting endocytosis and autophagy (3–6). However, C9orf72 knockout mice do not manifest motor neuron degeneration (7) and null-allele mutants have not been detected in ALS or FTD cases. Secondly, expanded G4C2 RNA transcripts may cause toxicity by forming RNA foci, which sequester RNA binding proteins, disabling the RNA processing machinery (8–10). Thirdly, repeat-associated non-AUG translation of G4C2 or G2C4 RNA can produce poly GA, poly GP, poly GR, poly PA and poly PR, which have been detected in the brains of *C9orf72* affected expansion carriers (11–13). Poly GA, poly GP and poly GR result from sense transcripts (14–16), whereas poly GP, poly PA and poly PR are translated from antisense transcript (17–19).

Recent studies provide evidence that DPR proteins are toxic. In several models, the arginine-rich DPRs poly GR and poly PR caused nucleolar stress, which has been suggested as a pathogenic mechanism in ALS and FTD (20,21). However, the contribution of poly GR and poly PR to human disease is controversial due to their scarcity in C9FTD/ALS brain tissue and their near absence in post-mortem spinal cord (22). In contrast, poly GA aggregation is frequently detected in C9FTD/ALS brain tissue and is associated with neurodegeneration in the frontal cortex (13). Poly GA is particularly aggregation-prone and generates predominantly neuronal cytoplasmic inclusions (NCI), which are also observed in urea fractions from C9FTD/ALS brain tissue (12). The expression of poly GA impairs neurite outgrowth and activates caspase-3 through endoplasmic reticulum (ER) stress (23). More recently, mouse models generated by AAV administration via the intracerebroventricular (ICV) route demonstrate that poly GA aggregates sequester the proteasomal degradation protein HR23 and impair nucleocytoplasmic transport proteins leading to neurodegeneration (24).

In this study, we generated vectors encoding 125 repeats of all five dipeptides from a non-G4C2 sequence and compared their toxicity to G4C2 expression in transfected human cell lines and chick embryo spinal cord. Our results show that poly GA is the most aggregation-prone and toxic of the five DPRs. Intriguingly, poly PA can suppress poly GA-mediated toxicity by inhibiting poly GA aggregation. Neurotoxicity in the chick spinal cord was comparable following G4C2 hexanucleotide and dipeptide electroporation, but we failed to detect any dipeptides from G4C2 repeat constructs, suggesting that both RNA foci and dipeptide repeats are contributing to the pathogenicity of the C9orf72 expansion in C9ALS/FTD. Poly PA could also suppress poly GA-mediated toxicity *in vivo*. This suggests an interplay between DPRs that should be considered as a facet of the underlying disease mechanism.

## Results

### G4C2 repeats cause neurotoxicity *in vivo*

**Figure 1.**
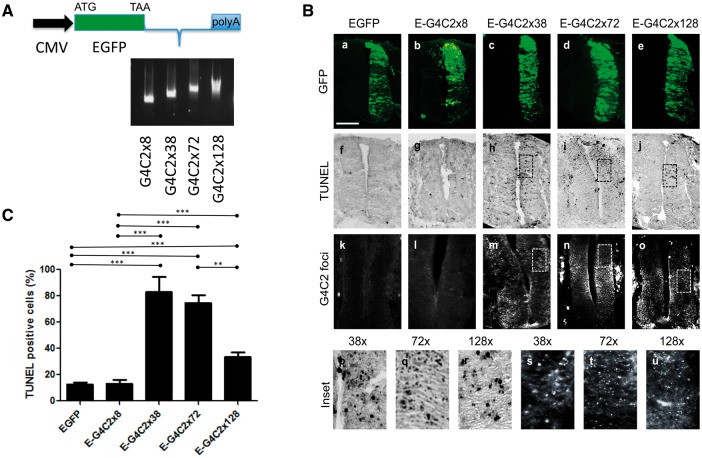
C9orf72 hexanucleotide repeats cause RNA foci and toxicity in the chick spinal cord. (**A**) Schematic diagram of the hexanucleotide repeat expressing constructs (G4C2x8, x38, x72, x128). (**B**) Transverse sections of chick embryo spinal cords at embryonic day 3.5 (E3.5) (dorsal at the top), electroporated with EGFP or EGFP-tagged hexanucleotide repeat constructs as illustrated (E-G4C2x8, x38, x72, x128). (a–e) Spinal cord sections were immunostained with anti-GFP antibody (*green*). (f–j) Apoptotic cell death was analysed by TUNEL staining (black). (k–o) G4C2 RNA foci detected by *in situ* hybridization (*white*). (p–r) Higher magnification images of corresponding TUNEL-stained sections (h–j). (s–u) Higher power images of corresponding panels *in situ* hybridized for RNA foci (m–o). Scale bar = 100 μm. (**C**) Quantification of TUNEL-positive cells (%), normalized to area of GFP fluorescence. Error bars represent the standard error for each sample (***P<0.0001). The data were analysed from six embryos in three independent sets of experiments.

In order to compare the toxic effects of dipeptide and G4C2 hexanucleotide repeats, we electroporated a range of expression constructs in the chick embryo. To determine whether expanded G4C2 transcripts show length-dependent toxicity *in vivo*, EGFP-tagged G4C2 repeat constructs of different repeat lengths (8x, 38x, 72x and 128x), were electroporated into the chick spinal cord at embryonic day 2.5 (E2.5). Since the cut-off repeat number for ‘risk’ is reported to be 30x (2), 8x is considered to be non-pathogenic and used as a negative control whereas the 38x, 72x and 128x repeats are considered potentially pathogenic (Fig. 1A). An EGFP construct was electroporated as a control. These constructs were designed with the capability to be transcribed but not translated through conventional AUG-initiated mechanisms. However, the possibility exists for translation into dipeptides using non-conventional ‘RAN’ translation so that both RNA-mediated and dipeptide-mediated toxicity could result. The electroporated embryos were re-incubated for 24 h until E3.5 and fixed, cryosectioned and immunostained with anti-GFP antibodies to determine the extent of construct expression (Fig. 1Ba–e). Terminal uridine nick-end labelling (TUNEL) was used to visualise apoptotic cells (Fig. 1Bf–j). We observed that EGFP and EGFP*-8x* electroporated embryos had very few TUNEL-positive cells on the electroporated side with no TUNEL-positive cells on the control side. Embryos electroporated with EGFP*-38x*, EGFP*-72x* and EGFP*-128x* constructs showed TUNEL-positive cells on the electroporated side. A smaller number of apoptotic cells were also detected on the non-electroporated side, suggesting that G4C2-mediated toxicity may spread contralaterally.

Toxicity was quantitated by measuring the area occupied by apoptotic cells as a percentage of the GFP-positive area for each section, to normalize for the efficiency of transfection. A mean percentage was then derived for each construct (Fig. 1C) under each condition. By this measure, embryos electroporated with EGFP*-38x*, EGFP*-72x* and EGFP*-128x* showed a marked increase in toxicity compared with either EGFP or EGFP*-8x*-expressing embryos. The EGFP*-38x* construct caused significantly higher levels of toxicity than the EGFP*-128x* construct. These results demonstrate that 38x repeats of G4C2 produce the most neuronal death in the chick embryo, with lesser amounts of cell death caused by 72x and 128x constructs.

### G4C2 hexanucleotide expansions form intranuclear RNA foci *in vivo*

One of the key RNA gain-of-function mechanisms associated with C9ALS/FTD neurodegeneration is the presence of RNA foci containing G4C2 repeats within neuronal nuclei in ALS and FTD patients. We analysed the prevalence of these RNA foci in chick embryos electroporated with G4C2 repeats using a Cy3-tagged G2C4 probe with fluorescent *in situ* hybridization. The G4C2-positive RNA foci were completely absent in the spinal cord sections from control EGFP, and EGFP-*8x* expressing embryos but were abundantly found in cells expressing the longer repeats; EGFP-*38x*, EGFP-*72x* and EGFP-*128x* (Fig. 1Bk–o). The RNA foci arising from EGFP-*38x* repeats were larger than those seen in EGFP-*72x* and EGFP-*128x* expressing embryos (Fig. 1Bs–u) and correlated with the higher prevalence of apoptotic cells seen with this construct.

### G4C2 hexanucleotide expansion repeats affect motor neurons

**Figure 2.**
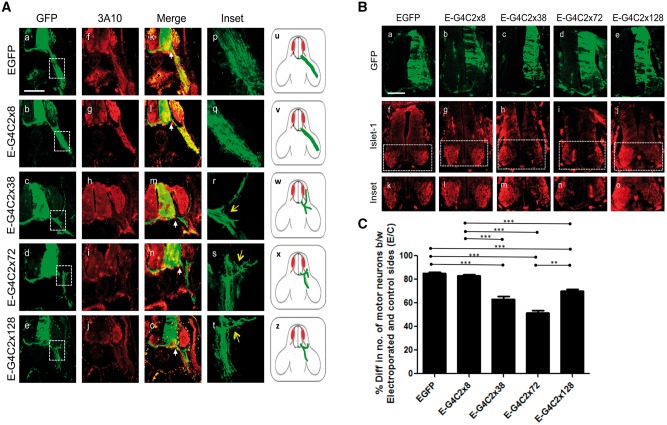
Hexanucleotide repeats affect motor neuron survival and motor axon pathways. (**A**) Transverse sections of E4.5 chick embryos, including spinal cord and periphery, immunostained for anti-GFP (a–e) and anti-neurofilament (f–j) to show electroporated motor axons on a background of normal nerve projections. (k–o) merged image between EGFP and 3A10 (neurofilament, red). Inset of boxed areas of the corresponding panels is magnified ten times in (p–t). Arrow indicates truncation and fasciculation of the axons (r–t). (u–z) Diagrammatic representation of perturbations of axon pathways (red; motor neuron, green; motor neuron outgrowth). (**B**) Embryos were electroporated with EGFP-G4C2x8, x38, x72, x128 at E3.5, then transverse sections of embryonic day E4.5 chick embryo spinal cords were immunostained (a–e) with GFP and anti-Islet-1 antibodies, which detect motor neurons (f–j). (**C**) The semi-quantitative analysis shows the percentage difference between the number of motor neuron on the control side and the number of motor neuron on the electroporated side. Scale bar=100 μm. The data were analysed from six embryos in three independent sets of experiments.

In order to determine any specific effects of the G4C2 hexanucleotide repeat expansions on motor neurons, we assessed the effects of G4C2 expression on motor axon projections 48 h post electroporation. E4.5, electroporated embryos were immunostained with antibodies to GFP and the anti-neurofilament-associated protein antibody (3A10) (Fig. 2A). EGFP control and EGFP*-8x*-expressing embryos were found to extend motor axons into the limb buds as expected and exhibited very little difference in the number of motor neurons between the control and the electroporated sides (∼ < 10%), suggesting negligible toxicity (Fig. 2B and C). Embryos expressing EGFP*-38x*, EGFP*-72x* and EGFP*-128x* repeats, however, showed evidence of abnormal nerve growth, including debundling/defasciculation and truncation as axon bundles, failed to extend into the periphery (Fig. 2A). Embryos electroporated with these constructs also had fewer motor neurons on the electroporated side compared to the control side (Fig. 2B and C), indicating that motor neurons are susceptible to the G4C2 hexanucleotide repeat-mediated toxicity which impairs the axonal outgrowth of surviving neurons.

### Dipeptide distribution in C9 human brain frontal cortex

**Figure 3.**
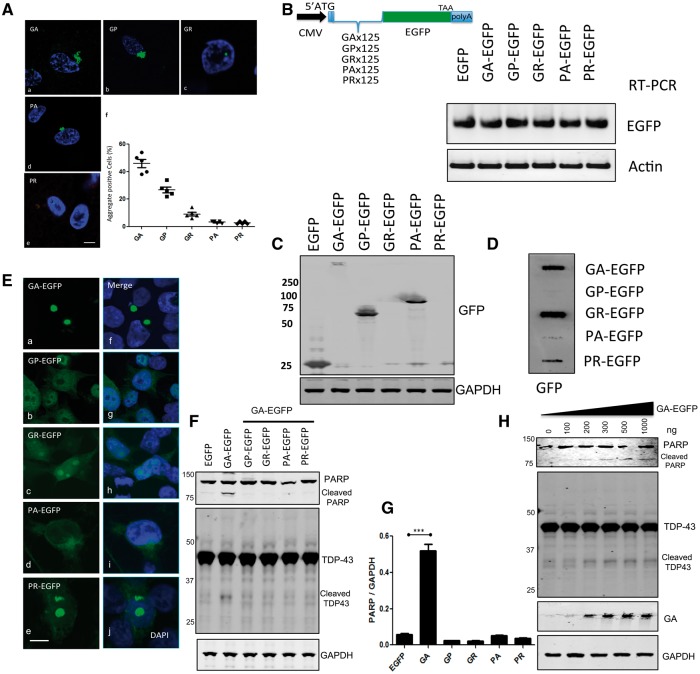
Poly GA is the most abundant DPR in human cortex and is toxic to cultured cells. (**A**) The total numbers of DPR-positive cells were counted in the *C9orf72*-positive human frontal cortex (n = 5). (a-e) Rabbit anti-DPR antibodies were used for the immunostaining for GA, GP, GR, PA, and PR (Green). DAPI (blue) was used for nuclear counterstaining, and (f) 50 cells were counted from each section (n = 5). Scale bar = 5 µm. (**B**) Schematic diagram of scrambled synthetic DPR, which incorporated start codon ATG. Artificially synthesized DPR DNAs were cloned into an EGFP expression vector, which generates N-terminal DPR-EGFP fusion proteins. Expression of DPR-EGFPs was tested in HEK-293 cells. Transcription of mRNA levels of transfected plasmids was assessed by semi-quantitative RT-PCR. (**C**) Western blot analysis of total cell lysates showed that poly GA forms a high molecular weight species. (**D**) Filter trap assay revealed that GA-EGFP, GR-EGFP and PR-EGFP are insoluble. An equal amount of lysates was loaded on nitrocellulose membranes. Membranes were stained with an anti-GFP antibody. (**E**) Expression of synthetic 125 repeat DPRs in HEK-293 cells results in the formation of cytoplasmic (GA-EGFP) or nuclear inclusions (GR-EGFP, PR-EGFP). In contrast, no inclusions were detected in GP-EGFP and PA-EGFP expressing cells. Scale bar = 5 µm. (**F**) Western blot analysis of total cell lysates from DPR-expressing cells for anti-PARP (top) and anti-TDP-43 (middle) revealed that GA-EGFP produced PARP cleavage, a marker of cell death and a 37 kDa TDP-43 cleavage product. GAPDH was used to normalize the protein loading. (**G**) Quantitative analysis of PARP western blotting showed that cells bearing inclusions of GA have significantly increased cleaved PARP (***P<0.0001). (**H**) Dose-response of GA-EGFP (50–10000 ng/well) was assessed for PARP (top) and TDP-43 (middle) cleavage. The high molecular weight of GA-EGFP (bottom) was increased following the dose of GA-EGFP plasmids.

To investigate the distribution of DPRs, we first sought to validate antibodies to GA, GP, GR, PA and PR and determine their abundance and distribution in human cortex samples (*n =* 5) (Fig. 3Aa–e). Immunofluorescence microscopy confirmed that poly GA, poly GP and poly GR were usually present within large cytoplasmic inclusions, whereas poly GR and poly PR dipeptides were detected within very small intranuclear inclusions. The percentage of DPR-positive inclusions in frontal cortex cells (*n =* 50 for each case) was poly GA (46%, 23 ± 3.0), poly GP (26.8%, 13.4 ± 2.1), poly GR (9.2%, 4.6 ± 1.4), poly PA (3.2%, 1.6 ± 0.5), and poly PR (2.8%, 1.4 ± 0.5) (Fig. 3Af). Thus, the most abundant DPR inclusions in the frontal cortex are derived from RAN translation of the sense strand transcript (GA > GP >GR). poly GP is also derived from the antisense strand and are far more common than those derived exclusively from the antisense strand (poly PA and poly PR). These results are consistent with our previous studies on the spinal cord samples of C9orf72 mutation-positive ALS cases (22).

### Characteristic features of dipeptide distributions *in vitro*

Neurotoxicity in *Drosophila* models caused by DPR proteins has been reported for the two arginine-rich DPRs poly GR and poly PR (20,21). Poly GA has also been suggested to cause neurotoxicity through the ER stress response (23) and the transport factor Unc119, which is linked to neuromuscular and axonal function, co-aggregates with poly GA (25). However, the other DPRs, such as poly GP and poly PA were not investigated. To investigate the toxicity of all five DPRs, we generated codon-scrambled constructs with an ATG translation initiation codon followed by a sequence encoding 125 dipeptide repeats tagged with EGFP at the C terminus (Fig. 3B). These constructs included a mixture of alternative codons with reduced GC content and lacking repetition to prevent the formation of RNA foci as is observed with repetitive G4C2-based constructs. In order to determine the stability of each codon-scrambled DPR RNA transcripts, we used semi-quantitative PCR and showed that the levels of EGFP mRNA expression were identical for all constructs (Fig. 3B). HEK-293 cells were transfected with DPRs, and after 48 h, we assessed DPR protein expression levels in HEK-293 cells by western blotting with the anti-GFP antibody (Fig. 3C). We found that the GA-EGFP protein was almost completely detergent-resistant and was retained at the top of the gel. GP-EGFP appeared at ∼75 kDa, and PA-EGFP was detected at ∼100 kDa, however, no GR or PR positive bands could be detected by western blotting. The failure of GR-EGFP and PR-EGFP detection could be due to the length of the positively-charged Arginine-rich peptide (125 repeats) which may prevent the protein from running through the gel (20). To resolve this issue, we used a filter trap assay, which confirmed that both GR and PR are efficiently expressed and can be detected as insoluble aggregates on the membrane (Fig. 3D).

We next sought to determine the subcellular distribution of GFP-tagged dipeptide proteins (Fig. 3E). GA-EGFP forms large cytoplasmic aggregates (Fig. 3Ea) and is occasionally found as aggregated form in the nucleus. The DAPI image shows that the GA-EGFP aggregates commonly indent a portion of the nucleus. GP-EGFP is distributed diffusely in both the cytoplasm and the nucleus (Fig. 3Eb) while PA-EGFP is predominantly in the cytoplasm (Fig. 3Ed). GR-EGFP and PR-EGFP are distributed predominantly within the nucleus and concentrated in granules (Fig. 3Ec and e), in a position consistent with a location in the nucleolus. Thus, DPR-EGFP constructs are expressed at equivalent levels, and their subcellular distribution within cells is consistent with previous reports (26,27). 

### Poly GA is the most toxic among the DPRs in transfected cells

In order to determine whether DPRs cause cellular toxicity, we have assayed the apoptosis marker poly (ADP-ribose) polymerase (PARP, 116 kDa), which produces an 89 kDa cleavage product in response to apoptosis. Of the five DPRs PARP cleavage (89 kDa) is only detected in GA-EGFP transfected cells (Fig. 3F and G), which agrees with the findings of Zhang et al., that 50 repeats of GA were able to activate apoptosis and caspase-3 activation (23). Remarkably, we also detected significant TDP-43 cleavage in GA-EGFP transfected cells (Fig. 3F), since TDP-43 cleavage is a biochemical signature in ALS and FTD (28). Based on these results, we speculate that GA toxicity may be dependent on the amount of protein expressed. To test this hypothesis, we transfected HEK-293 cells with a range of GA-EGFP plasmid concentrations (50–1000 ng per 40, 000 cells) and demonstrate that PARP and TDP-43 cleavage is GA-dose responsive, increasing in parallel to the appearance of high molecular weight GA-EGFP aggregates on western blot (Fig. 3H). In this cellular model, we did not observe toxicity from GR-EGFP and PR-EGFP expression, which suggests that some cell lines may be resistant to the toxic effects of these DPRs.

### Dipeptides cause toxicity in embryonic chick spinal cord

**Figure 4.**
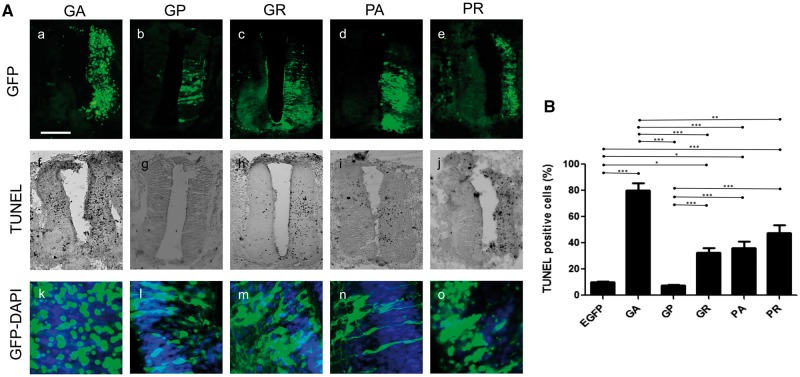
Poly GA is most toxic DPR in chick spinal cord. (**A**) Transverse sections of embryonic day 3.5 (E3.5) chick embryo spinal cords electroporated with DPR constructs as labelled (GA, GP, GR, PA and PR) with 4 µg of plasmid, which causes toxicity in the chick spinal cord. (a–e) Expression of DPR detected with GFP Immunostaining on sections of electroporated spinal cord. (f–j) Cell toxicity of DPRs were assessed by TUNEL assay. (k–o) Merge image of DAPI and DPR. Scale bar = 100 μm. (**B**) Quantitative analysis of TUNEL-positive cells showing that cells bearing inclusions of GA, PR have significantly increased TUNEL positive staining (***P<0.0001).

To investigate dipeptide-mediated neurotoxicity *in vivo*, we expressed the five scrambled-codon dipeptide constructs in the chick spinal cord. Each DPR construct was electroporated into the embryonic chick spinal cord, and the embryos were re-incubated for 24 h. The embryos were then cryosectioned and immunostained with anti-GFP antibodies, to determine the extent of DPR expression, and TUNEL-labelled to assay apoptotic cell death. Differences were observed between the dipeptides in the dorsoventral extent of transfection in the spinal cord (Fig. 4Aa–e) and in the subcellular distribution of DPR proteins (Fig. 4Ak–o). For example, GA-EGFP and PR-EGFP expression appeared to form aggregates (Fig. 4Aa and k), whereas GP-EGFP, GR-EGFP and PA-EGFP were expressed diffusely throughout the neuronal cytoplasm (Fig. 4Al–n). Differences in DPR regional localization meant that it was impossible to normalize toxicity according to the EGFP area in any single section, so manual counting of TUNEL and GFP-positive cells was used instead. Embryos electroporated with EGFP alone showed very little toxicity, whereas embryos electroporated with GA-EGFP showed the highest level of toxicity that was significantly different from the control (*P <* 0.0001; Fig. 4B). Of the other constructs, there was toxicity in a descending order PR > PA > GR (Fig. 4B), however GP-EGFP showed negligible toxicity.

### Poly GA sequesters other dipeptides

**Figure 5.**
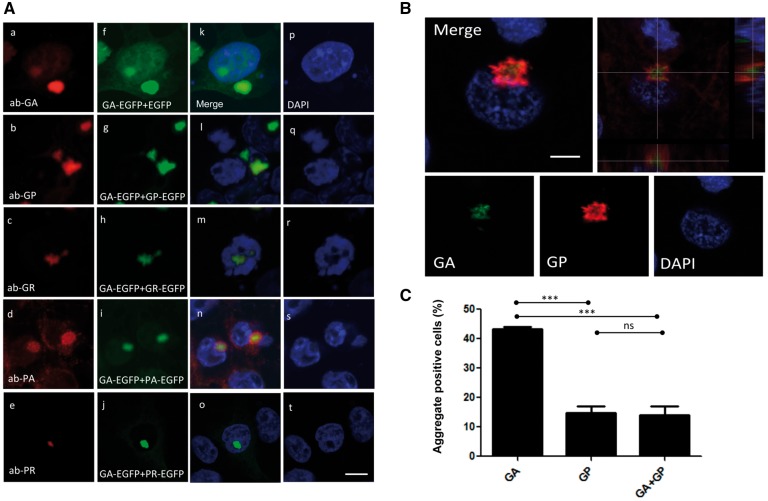
Crosstalk between DPR proteins: GA sequesters GP. (**A**) HEK-293 cells were transfected with (GA-EGFP+EGFP, GA-EGFP+GP-EGFP, GA-EGFP+GR-EGFP, GA-EGFP+PA-EGFP and GA-EGFP+PR-EGFP). Cells were immunostained with specific rabbit antibodies against DPRs (GA, GP, GR, PA and PR; red). Green indicates EGFP-expressing DPRs and blue shows nuclear counterstaining with DAPI. Scale bar = 5 μm. (**B**) Human brain sections (temporal lobe) from C9orf72 expansion carriers were used for double-staining with poly GA (green) against poly GP (red) (*n*=3). (**C**) The percentage of poly GA and poly GP positive cells show that the majority of poly GP aggregates are positive with poly GA on hippocampal neuron (***P<0.0001).

Both sense and antisense RNA foci have been found in the same cell (27,29). Thus all five dipeptides have the capability of being expressed in a single cell. Here we demonstrated that poly GP and poly PA did not aggregate in transfected cells (Fig. 3Eb and d), but both are found as aggregates in the brain of c9orf72 positive cases (Fig. 3A). Therefor, we sought to test the hypothesis that certain DPRs may affect the behaviour of others regarding their localization, aggregation and toxicity. As poly GA is the most aggregate-prone, we co-transfected GA-EGFP with other dipeptides (GA + GP, GA + GR, GA + PA, GA + PR), and immunostained cells for the different dipeptides and found that GA can co-localise with the other DPRs (GP and PA) (Fig. 5A). In order to assess whether such co-localization is detectable in human brain, we performed double immunostaining with anti-GA and anti-GP antibodies. We observed that GA was localized in the core of an inclusion surrounded by GP (Fig. 5B) in 10% of cells containing GA inclusions (Fig. 5C). Overall, results indicate that GA is a source of seeding protein, which sequesters GP.

### Poly PA inhibits PARP cleavage and aggregation of poly GA

**Figure 6.**
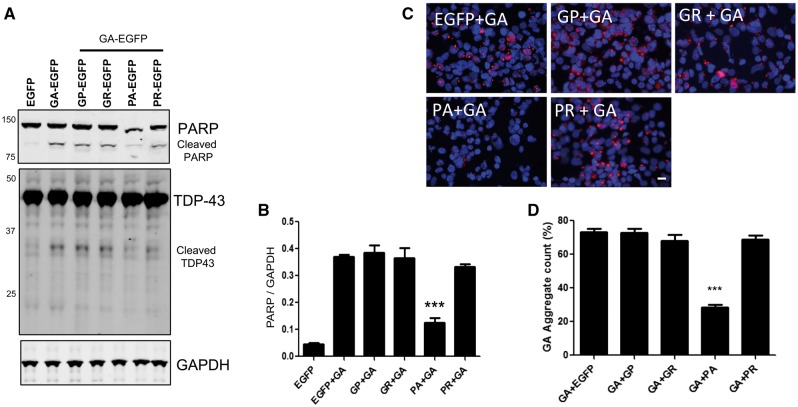
Poly PA inhibit PARP cleavage and aggregation of poly GA. (**A**) Dual transfection of GA-EGFP with PA-EGFP decreases PARP cleavage. GA-EGFP (250 ng) with GP-EGFP, GR-EGFP, PA-EGFP and PR-EGFP (250 ng) were transfected in HEK-293 cells, and total cell lysates were used for PARP and TDP-43 (middle) analysis by western blotting. (**B**) Quantitative analysis of cleaved PARP showing that cells bearing inclusions of GA+PA have significantly decreased levels of cleaved PARP (***P<0.0001). (**C**) Dual (DPR-EGFPs + GA-EGFP) transfected HEK-293 cells were stained with anti-GA antibodies (red). Blue represents nuclear counterstaining of DAPI. Scale bar = 10 μm. (**D**) Quantitative analysis of GA-EGFP positive cells showing that cells are bearing inclusion of GA, PA have significantly decreased GA-EGFP aggregates (***P<0.0001).

We further speculated that GA-dependent toxicity might be altered by the presence of other dipeptides, which we then tested by dual transfection. Interestingly, among the dipeptides, co-transfection with GA-EGFP and PA-EGFP reduced PARP cleavage by ∼50% compared with GA-EGFP and other dipeptides (Fig. 6A and B). In addition, the 35 kDa cleaved TDP-43 fragment was decreased by 50% in double transfected (PA-EGFP with GA-EGFP) cells. Then, we tested the number of GA aggregates in the presence of other dipeptides using GA specific monoclonal antibody (Fig. 6C). There was a sharp reduction in the numbers of GA-positive aggregates in co-transfected cells (*P <* 0.0001) (Fig. 6D).

### Poly PA inhibits poly GA toxicity in chick spinal cord

**Figure 7.**
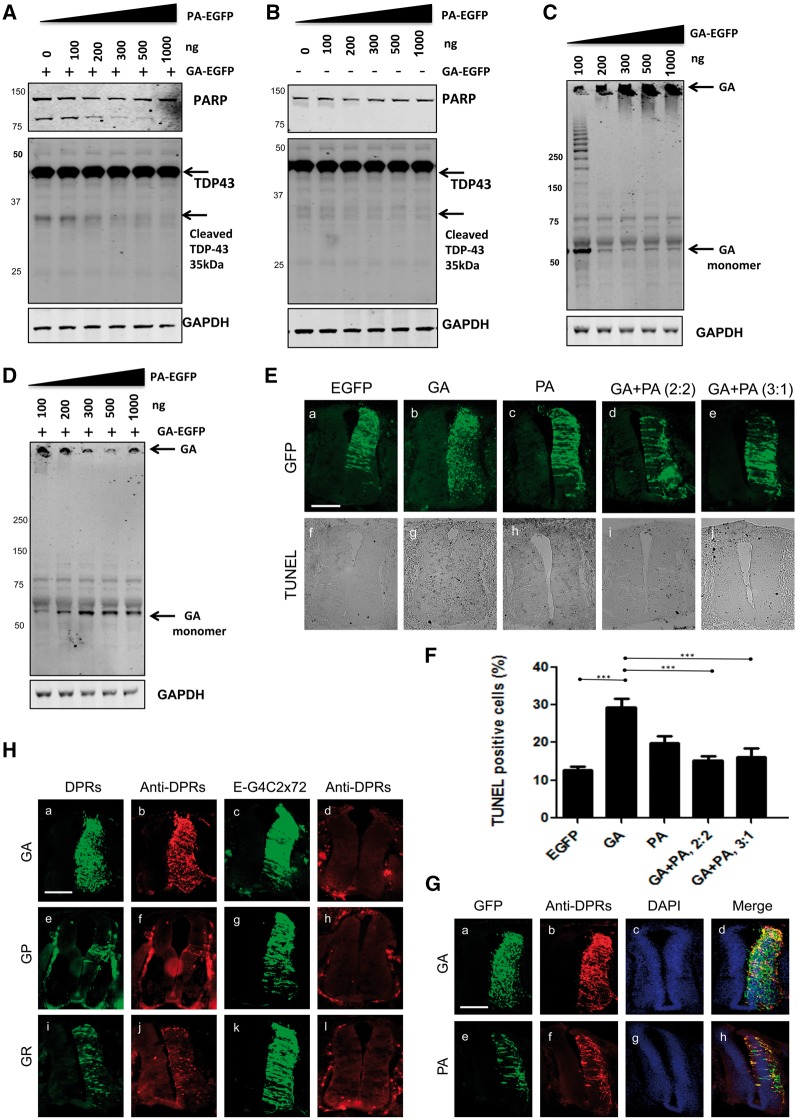
Poly PA inhibits poly GA toxicity in chick spinal cord: DPR versus RNA toxicity of G4C2 hexanucleotide repeats. (**A**) Cleaved PARP and TDP-43 were assessed in lysates, of HEK-293 cells co-transfected with GA (250 ng) and increasing concentrations of PA-EGFP (0-1000 ng). (**B**) PA-EGFP expression did not increase PARP cleavage. Increasing concentrations of PA-EGFP (0-1000 ng) were transfected without GA-EGFP. (**C**) High molecular weight GA-EGFP species is increased in a dose-response manner. Total cell lysates from GA-EGFP (50–1000 ng) transfected HEK-293 cells were used for anti-GA western blotting. (**D**) PA-EGFP inhibits GA-EGFP aggregation. The appearance of high molecular weight GA-EGFP species is inhibited by a gradual increase of PA-EGFP. (**E**) PA-EGFP rescues GA-EGFP- mediated toxicity in the chick spinal cord. Transverse sections of embryonic day 3.5 (E3.5) chick embryo spinal cords (dorsal at top), electroporated with DPR constructs as labelled – GA and PA embryos were electroporated with 2 µg of plasmid. GA-EGFP: PA-EGFP (2: 2) and GA-EGFP: PA-EGFP (3: 1) were electroporated with different concentrations (µg) of plasmids as indicated. Scale bar = 100 μm. (**F**) Quantitative analysis of TUNEL-positive cells showing that the number of TUNEL- positive GA-EGFP expressing cells are significantly decreased by PA-EGFP expression in chick spinal cord (***P<0.0001). (**G**) GA-EGFP and PA-EGFP expression in chick spinal cord were detected by immunostaining for GA and PA. Scale bar = 100 μm. DAPI and GFP show subcellular localization of DPRs by using DPR antibodies. Scale bar= 100 μm. (**H**) Comparison of DPR expression between synthetic DPR construct and G4C2 repeat construct by DPR immunostaining in chick spinal cord. The GA-EGFP, GP-EGFP, and GR-EGFP constructs (a, e, i) are transfected with the same amount of EGFP-G4C2x 72 repeat construct (c, g, k). DPR expression levels were compared with specific poly GA, poly GP and poly GR antibodies. Scale bar = 100 μm. The data were analysed from six embryos in three independent sets of experiments.

In order to understand the role of PA in GA toxicity, the amount of PA-EGFP plasmid used for transfection was gradually increased while keeping a constant expression of GA-EGFP and PARP cleavage was monitored. We found that PARP cleavage was dramatically reduced by high levels of PA (Fig. 7A). To ensure that PA-EGFP itself has an effect on PARP cleavage, a similar dose response of PA-EGFP transfection was performed without GA-EGFP, which resulted in no PARP cleavage being detected (Fig. 7B). To further investigate the biological relevance of these phenomena, we tested the effect of increasing GA-EGFP expression on its propensity to aggregate. We found that transfection of 100 ng of DNA transfection produced monomeric GA-EGFP, with a molecular weight of around 60 kDa and showing multiple bands of GA throughout the gel from 100 ng of GA-EGFP (Fig. 7C). However, on transfecting 200 ng of GA-EGFP led to complete aggregation of the DPR that remained on top of the gel. In contrast, double transfection with increasing concentrations of PA-EGFP with GA-EGFP reduced the amount of aggregated GA-EGFP trapped on top of the gel and increased the level of monomeric GA-EGFP (Fig. 7D). These results suggest that PA-EGFP accumulation inhibits GA-EGFP aggregation and toxicity.

In view of the ability of poly PA to rescue poly GA toxicity *in vitro*, we also tested this idea *in vivo* by electroporating chick spinal cord with both GA-EGFP and PA-EGFP plasmids at 2 µg: 2 µg and 3 µg: 1 µg of GA-EGFP: PA-EGFP dosages in order to compare their toxicity using the TUNEL assay (Fig. 7Ea–e). We observed a significant toxic effect with GA-EGFP alone, (Fig. 7Eg) albeit the incidence of cell death was lower than the following electroporation with twice the plasmid concentration (Fig. 4A).

Dual transfection with either GA-EGFP + PA-EGFP (2: 2) or GA-EGFP + PA-EGFP (3: 1) resulted in a more diffuse GFP signal throughout the cell, an absence of focal aggregates and a significant reduction in the number of TUNEL-labelled cells (Fig. 7Ei, Ej and F). These results suggest that expression of PA-EGFP with GA-EGFP in the chick spinal cord produced a dose-dependent reduction of GA-mediated toxicity (Fig. 7F). Immunohistochemistry using anti-GA and -PA specific antibodies confirmed expression of both DPR (Fig. 7G).

### DPR versus RNA toxicity of G4C2 hexanucleotide repeat

In this study, we have shown that the expression of both G4C2 hexanucleotide repeats and scrambled alternative-codon DPRs are both neurotoxic to differing degrees in cellular and *in vivo* models, but it is not clear which of these mechanisms is likely to predominate in ALS and FTD. In order to determine whether DPR expression contributed to G4C2 toxicity in the chick spinal cord, we used dipeptide-specific antibodies to compare the expression of DPRs and G4C2 72x repeats. All of these antibodies were able to detect DPR inclusions in C9orf72 patient tissues and chick spinal cords electroporated with scrambled constructs (GA-EGFP, GP-EGFP and GR-EGFP) (Fig. 7Hb, f and j), however, immunostaining on the sections from EGFP-G4C2x72 repeats failed to yield any DPR staining (Fig. 7Hd, h and l). This result indicates that the neurotoxicity observed in the chick spinal cord following electroporation of G4C2 repeats must result purely from an RNA-based mechanism and not from RAN translation. Overall these results suggest that toxicity can arise from either G4C2 RNA or dipeptide repeats, with poly GA the most toxic of the DPR isoforms in our cellular and animal models.

## Discussion

Since the hexanucleotide repeat expansion was discovered in the first intron region of the *C9orf72* gene, several major hypotheses have emerged to explain its toxic mechanism, ranging from haploinsufficiency, RNA foci formation and DPR-mediated toxicity. The discovery of DPRs has fueled research not only on C9 but also the repeat-related diseases such as Fragile X-associated tremor ataxia syndrome (FXTAS) and myotonic dystrophy (DM1), which opens up a new aspect of the pathology of neurodegenerative disease (30,31). As DPRs have become a key area of investigation in *C9orf72*-mediated pathology, we have investigated the potential toxicity of RNA and DPRs using the chick embryo as an *in vivo* model.

Here we show that C9orf72 repeat constructs produce toxicity in the chick spinal cord, consistent with our previous, data in the zebrafish model (10). However, in this case, we found that the toxicity did not correspond to the length as the G4C2x38 repeat showed 50% more toxicity than G4C2x128 (Fig. 1C). We have previously shown that G4C2x72 repeats were less toxic than G4C2x38 repeats in SH-SY5Y cells, which is consistent with our chick embryo model (10). In addition, we have shown that 38x repeat constructs produce the largest number of RNA foci, consistent with the large amount of cell death of the constructs tested.

In order to investigate the effects of dipeptide-mediated toxicity, we surveyed the expression patterns of all of the DPRs in human post mortem tissue sections from the frontal cortex of C9orf72 expansion carriers and discovered aggregated forms of poly GA, poly GP and poly PA in the cytoplasm whereas the poly GR and poly PR was detected mainly in the nucleus. Intriguingly, among the DPRs, poly GA was the most frequently found in the frontal cortex, which is consistent with other reports (13) and was the most aggregate-prone and toxic in our cellular model (Fig. 3E and C). Poly GA was the only DPR, which had the potential to produce PARP cleavage, a marker of apoptosis, in a dose-dependent manner. TDP-43 cleavage, a key feature of ALS pathology, also resulted from poly GA over-expression. Arginine-rich dipeptide poly GR or poly PRs have been reported as toxic (20,21); currently we cannot rule out the relevance of the poly GR and poly PR toxicity in our cell model, even though we found their prevalence to be less than that of poly GA in the brain. Due to the fact that PARP cleavage has only been found cells expressing poly GA, which is correlated with TDP-43 cleavage, we can conclude that the most toxic DPR is poly GA, at least in our cell culture system.

Moreover, *in vivo* injection of the DPR plasmid into the chick embryonic spinal cord demonstrates that the most toxic species was poly GA compared with other DPRs (Fig. 4A). These results match with our cellular data that GA is the most toxic among the DPRs. There are several reports of the pathological role of poly GA, suggesting that aggregated poly GA leads to ER stress, which activates apoptosis in primary neurons (23). In contrast, poly GA aggregates have been suggested to act as protective granules which sequester toxic components in the cytoplasm, enabling cells to sustain during stress (32). In this case, it is important to compare these results with similar aggregating protein poly-glutamine (polyQ) diseases, which resemble GA toxicity (33) in many ways. Firstly, polyQ aggregates of large inclusions are proposed as a proximal cause of neurodegeneration (33). Secondly, the expanded polyQ protein is aggregate-prone and the expanded poly-glutamine track facilitates the transition to toxic conformation. Finally, it could cause protein-misfolding stress such as autophagy or UPR. These routes need to be addressed in future studies.

Although the toxicity of DPRs is still debated, all DPRs have been found as aggregated forms in human brain, with occasionally diffuse distribution detected. We have noticed that the single expression of poly GP or poly PA in a cell culture model did not show aggregation, as detected in human brain. However, when expressed together with poly GA, poly GP or poly PA were able to form aggregates. The co-expression study of poly GA with other DPRs demonstrates co-localization within cells, raising the possibility that poly GA ‘seeds’ DPR aggregation. We have found that the poly GA was at the core of the aggregate of poly GP aggregates in the human brain. Mackenzie et al., demonstrated that cytoplasmic poly GA was found in the centre of the cell being surrounded by aggregated TDP-43 in dentate granule cells, which is similar to what we have found with poly-GP (12). Therefore, there might be more preference by those DPR interactions with poly GA (Fig. 5A).

While characterizing the toxic effects of DPRs, we found that poly PA protected PARP cleavage (Fig. 6A and B). We therefore further speculate that poly PA is able to decrease poly GA aggregation. We confirmed that the poly GA aggregation was decreased by about 40% following poly PA overexpression, which suggested that poly PA inhibits poly GA aggregation. The biochemical analysis showed that poly PA inhibits poly GA aggregation in a dose-dependent manner. These results may suggest that poly PA is able to compete with poly GA for aggregation. The double injection of poly GA and poly PA in the chick model demonstrated similar results that poly PA decreased poly GA toxicity.

We have previously used the chick embryo to model the pathological effects of TDP-43 wild-type and mutant constructs. We found that TDP-43 mutants caused neurotoxicity, accompanied by striking defects in axon outgrowth, implying that cytoskeletal dysregulation may play a role in ALS and FTD-TDP pathogenesis. Similar phenotypes were observed following over-expression of wild-type and mutant TDP-43 (34), suggesting that a common mechanism might link *C9orf72* and TDP-43 to ALS and FTLD. In addition, co-expression of poly PA with poly GA could rescue poly GA-mediated toxicity *in vitro* and *in vivo* (Fig. 7), further suggesting the capacity for DPRs to interact with one another. Finally, a comparison of RNA-mediated and DPR-mediated toxicity in the chick spinal cord *in vivo* suggested that are both toxic mechanisms in this *in vivo* model. These results may suggest that the pathology of DPR is more complicated than our assumption from individual C9 RNA studies.

Overall, here we demonstrate that over-expression of G4C2 hexanucleotide expansion repeats and in particular of repeat lengths of 38x confer neurotoxicity in the chick model. The G4C2 hexanucleotide repeat (38x, 72x and 128x) in particular, forms RNA foci, affecting motor neurons and motor axon projections. Although it was difficult to compare directly because of differences in expression patterns, we found that dipeptide-encoding constructs caused similar levels of toxicity as G4C2 repeats. Importantly, in our chick model, the expression of G4C2 repeats did not result in DPRs, suggesting that RNA repeat expansion mediated toxicity is not through the DPRs. Therefore, the toxicity of RNA foci and DPRs are not mutually exclusive and both contribute to the pathomechanism of C9orf72 linked disease.

## Materials and Methods

### Dipeptide cloning

Poly GA, poly GP, poly GR, poly PA, and poly PR (125 repeats) sequences were synthesized by GeneArt (Life Technologies). The protein sequences of each dipeptides were uploaded on the web-based Geneart program and converted to DNA sequence using a codon optimization process which generates random DNA sequences of each dipeptides. All plasmid constructs were re-sequenced and sized later in the experiments to ensure that there were no rearrangements (Supplementary Material, Fig. S1). The HA tag was included 5’ of GA and GP. The synthesized C9RAN DNAs were used for cloning into a lentiviral shuttle vector which expressing EGFP. The plasmids were used for *in vitro* and *in vivo* transfection study.

### Hexanucleotide repeat plasmid constructs

The details of EGFP plasmids containing the GGGGCC hexanucleotide repeat sequence of C9orf72 (G4C2) with 8x, 38x, and 72x repeats were described previously (10). We have further cloned 128x repeat into the 5’ EGFP vector. These constructs, driven by a CMV promoter, were used for chick electroporation experiments. pEGFP-C1 expression vector was used to express EGFP as described previously (34).

### Cell culture

HEK-293 cells were maintained in Dulbecco’s modified Eagle medium (DMEM; Life Technologies) supplemented with 10% feotal bovine serum (Life Technologies), and maintained at 37 °C, 5% CO_2_. Cells were plated a day before transfection and the medium was replaced with fresh medium before transfection using Lipofectamine 3000 (Life Technologies).

### Western blotting and immunofluorescence

Five to ten µg of cell lysate from the RIPA fraction and the equivalent liquid volume from the fraction were loaded on gels. Western blot quantification was performed using ImageJ. Integrated band intensities were normalized to that of loading control or the RIPA fraction. Cells were fixed in 4% PFA (Sigma) at 4 °C for 20 min and washed with PBS three times for 10 min. Cells were then permeabilized using PBS containing 0.1% TritonX-100 (Sigma) for 5 min at room temperature with gentle shaking, followed by blocking in PBS containing 1-5% donkey serum for 1 h at room temperature. Cells were then incubated with primary antibody diluted in blocking solution overnight at 4 °C. After washing with PBS, cells were incubated with fluorescent secondary antibodies (1: 500) diluted in blocking solution for 1 h at room temperature. DAPI (Sigma) was used for staining of nuclei and the slides were mounted in FluorSave (Calbiochem). To detect insoluble C9RANs, a filter trap assay was performed. Cells were lysed in RIPA buffer and the protein concentrations were assessed by DC protein assay kit (Bio-rad; 5000111). Total proteins (2 μg/100µl) were filtered through a cellulose acetate membrane (pore size 0.2 μm) then washed three times according to a published protocol (36). Aggregates retained on the filter were detected with an anti-GFP antibody.

### Human tissue processing

All cases were provided by the MRC London Neurodegenerative Diseases Brain Bank (Institute of Psychiatry, Psychology and Neuroscience, King’s College London, UK) and were collected and distributed according to local and national research ethics committee approvals (Supplementary Material, Table S1 for case details). Sections from the frontal cortex were provided as 10% formalin-fixed and paraffin-embedded blocks. To perform double immunostaining, paraffin was removed with xylene and sections were rehydrated in an ethanol series (100, 95, 70%) for 3 min each. Slides were incubated in 0.3% Sudan black for 5 min to quench autofluorescence and washed with water. Antigen retrieval was carried out by microwaving for 6 min at maximum power and 12 min at medium power in 100 mM sodium citrate buffer (pH 6.0). Non-specific binding sites were then blocked for 20 min using 5% normal donkey serum in PBS.

For immunofluorescence staining, sections were incubated with primary rabbit antibodies against the five different DPRs (poly-GA, GP, GR, PR and PA) at a dilution of 1: 100 or monoclonal GA (Millipore MABN889) at 1: 100 overnight in a humid chamber at 4 °C. The C9RAN antibodies were previously characterised (23). After three washes with PBS, sections were incubated with anti-rabbit (Dylight 594) and anti-mouse (Dylight 488) secondary antibodies for 1 h at room temperature. DAPI (Sigma) was used to counterstain nuclei. Sections were mounted in Fluorsave. Semi-quantitative and quantitative evaluation of C9RANs was performed using a Zeiss Axiovert S100 microscope. Aggregates were also imaged using a Leica Confocal SP microscope. The number of DPR aggregates was counted from fifty DAPI positive cells per case. Statistical analysis was performed with GraphPad Prism software (version 5.0).

### Chick embryo preparation

Fertilised Brown Bovan Gold Hen’s eggs (Henry Stewart & Co, UK) were incubated in a forced draft incubator at 38 °C for 2.5 days (HH14-15) and staged (37). All embryos were treated in accordance with the Animals (Scientific Procedures) Act of 1986, UK.

### *In* *o* *vo* electroporation

Expression of constructs was achieved by *in ovo* electroporation as described (34,38). Genes of interest were expressed in one- half of the spinal cord. Briefly, eggs were windowed, and Tyrode’s solution containing 1% penicillin–streptomycin (Invitrogen) was added. Plasmid DNA constructs were dissolved in ddH_2_O (1–4.0 μg/μl with 0.5% fast green) and pressure-injected into the lumen of the spinal cord. Platinum electrodes were positioned in parallel on either side of the spinal cord, and a square pulse at 20 mV was applied five times for 50 ms at 1 s intervals. In all the experiments, *pEGFPC1* expression vector was used as a control. Eggs were resealed and incubated for 24–96 h, after which surviving embryos were dissected and fixed using 4% paraformaldehyde for 1–2 h at room temperature.

### Chick embryo immunofluorescence

Fixed embryos were washed with PBST (PBS (Invitrogen) + 0.1% Triton X-100 (Sigma)) three times and processed through increasing concentrations of sucrose into OCT, snap-frozen and sectioned at 20 μm. Frozen sections were washed in PBS, permeabilised using PBST (1 × PBS + 0.5% Triton X-100). Sections were then blocked using 10% FCS/PBS for 1 h and incubated with primary antibodies overnight at 4 °C (mouse anti-islet1/2 (4D5), 1: 200, DSHB; mouse anti-neurofilament associated protein (3A10), 1: 200, DSHB; chicken anti-GFP, 1: 1000, Abcam). Anti-dipeptide antibodies were applied as for human tissue.

Sections or cells were washed again with PBS, three times for 5 min each, before incubation with fluorescent secondary antibodies for 1–2 h at room temperature (anti-rabbit Alexa Fluor 488; anti-mouse Alexa Fluor 568; anti-mouse Alexa Fluor 488, anti-chicken Alexa Fluor 488, all 1: 1000, all Invitrogen). After further washing, sections or cells were mounted using Hard-set Vectashield with DAPI (Vector Laboratories). Images were captured using a Zeiss Axioskop microscope with Volocity software.

### TUNEL toxicity assay

To assess toxicity, the DeadEnd colourimetric TUNEL (Promega) system was used to assay apoptotic cells in tissue sections. Only sections with more than 50% transfection (as detected by the GFP staining) were used for the assay.). For the hexanucleotide repeat constructs, the GFP area used for a good indicator of the extent of electroporation, the area occupied by TUNEL-positive cells were quantified using Fiji software, and expressed as a percentage of the area of GFP-expression in each section (quantified in the same way). For DPR constructs, expression patterns differed markedly between constructs and could not be standardized according to GFP staining. Therefore, apoptotic nuclei were manually counted in sections judged to have sufficient electroporation (<50% area). For RNA repeats, data were grouped from 42 sections across at least six embryos per condition from three experiments, and for DPR constructs, data were analysed from 35 sections across at least six embryos from three experiments. A mean percentage was calculated across groups and compared statistically using a one-way ANOVA coupled with Dunn’s multiple comparison tests.

### Fluorescent *in situ* hybridization

Cy3-labelled G2C4 ×8 RNA probes were synthesized by Integrated DNA Technologies and used as antisense probes. Sections were fixed with 4% DEPC PFA for 15 mins, washed twice with 1x DEPC PBS, permeabilized with 0.3% Triton X-100 (Sigma-Aldrich) in 1x DEPC PBS for 20 min and equilibrated in 1x SSC with 40% formamide for 5 min. The sections were pre-hybridized in 200 µl (per slide) of hybridization solution (50% formamide, 5x SSC, 5x Denhardts, 0.4 mg/ml Torula yeast RNA, 0.1 mg/ml of baker’s yeast) for 2 h at 65 °C in humidified chambers. The probes (10 ng/µl) were added to the hybridization solution and heated at 95 °C for 5 min. Hybridization was performed overnight at 65 °C in humidified chambers. The following day, sections were washed twice with 0.5x SSC, 40% Formamide and 0.1% Tween-20 solution for 30 min at 65 °C, then washed 3x with PBS-T (1x PBS + 0.1% Triton X-100) and fixed with 4% PFA for 5 min. For immunostaining with FISH, sections were blocked with 1% BSA in PBS-T for 1 h, and then sections were incubated with primary antibodies as above. Nikon fluorescence and confocal systems were used for high-resolution imaging.

### Semi-quantitative scoring of RNA foci

For electroporated chick embryonic spinal sections, the numbers of RNA foci on the transfected side of the spinal cords were manually counted, using only sections with more than 50% cells transfected (as detected by GFP staining). The data were analysed from ∼20 sections across at least three embryos from three sets of experiments. Data were therefore binned as sections with 100 or less foci, 100–200 or 200–300. 

### Statistics

Statistical analysis was carried out using GraphdPad Prism. Unpaired two-tailed Mann–Whitney *T*-test was used to compare the abundance of TUNEL-stained cells in chick embryos. Data are presented as mean ± SEM. Statistical significance was set at *P <* 0.05.

## Supplementary Material

Supplementary Material is available at *HMG* online.

## Supplementary Material

Supplementary Data
