# Supplementary material for: C9orf72 poly GA RAN-translated protein plays a key role in amyotrophic lateral sclerosis via aggregation and toxicity
Source: Hum Mol Genet. 2017 Sep 13;26(24):4765–77. doi: 10.1093/hmg/ddx350 (PMC5886201; doi:10.1093/hmg/ddx350)
Supplement: Supplementary Data [file ddx350_hmg-supplemental-revision-160817.docx]

**Supplementary material**

**C9orf72 poly GA RAN-translated protein plays a key role in Amyotrophic Lateral Sclerosis via aggregation and toxicity**

**Classification: Biological Science: Cell biology**

- Youn-Bok Lee^1*^, Pranetha Baskaran^2*^, Jorge Gomez^1^, Han-Jou Chen^1^, Agnes Nishimura^1^, Bradley Smith^1^, Claire Troakes^1^,Yoshitsugu Adachi^1^, Alan Stepto^1^, Leonard Petrucelli^4^ , Jean-Marc Gallo^1^, Frank Hirth^1^, Boris Rogelj^3^, Sarah Guthrie^2^, Christopher E Shaw^1^†

^1^United Kingdom Dementia Research Institute Centre, Maurice Wohl Clinical Neuroscience Institute, Institute of Psychiatry, Psychology and Neuroscience, King’s College London, 125 Coldharbour Lane, Camberwell, SE5 9NU London, U.K. ^2^Department of Developmental Neurobiology, King’s College London, Guy’s Campus, London SE1 1UL, UK. Department of Biotechnology, Jožef Stefan Institute, Jamova 39, SI-1000 Ljubljana, Slovenia^3^.  Department of Neuroscience, Mayo Clinic Florida, Jacksonville, FL 32224, USA^4^

- * These authors contributed equally
- † Corresponding author: Christopher E Shaw
- Department of Basic and Clinical Neuroscience,
- [christopher.shaw@kcl.ac.uk](mailto:christopher.shaw@kcl.ac.uk)

**Table S1.**

| **Case** | **Age** | **Sex** | **PMD (hours)** | **Age of Onset** |
| --- | --- | --- | --- | --- |
| **ALS (*C9ORF72*)** | 39 | F | 70 | 35 |
| **ALS/FTLD (*C9ORF72*)** | 43 | F | 69 | 42 |
| **ALS/FTLD (*C9ORF72*)** | 62 | M | 74 | 61 |
| **FTLD-U (*C9ORF72*)** | 79 | M | 35 | 68 |
| **ALS/FTLD (*C9ORF72*)** | 53 | M | 82 | 52 |
| **Control** | 43 | F | 43 | N/A |
| **Control** | 67 | M | 41 | N/A |
| **Control** | 90 | F | 50 | N/A |
| **Control** | 57 | M | 26 | N/A |
| **Control** | 74 | M | 23 | N/A |

**Table S1. Human case details which used for immunostaining**

- Age, Sex, post-mortem delay (PMD) and age of onset are indicated in the table.

1. **GA**

atggcatacccatacgacgtcccagactacgccggagctggcgcaggcgctggggcaggggctggcgccggggccggggccggcgctggggctggcgcaggggcaggcgctggcgcaggcgcaggggctggggcaggcgctggcgctggcgctggcgcaggcgctggggctggcgctggggcaggggcaggggcaggcgcaggggctggcgctggcgctggggctggggccggggccggcgcaggcgccggggccggggccggggcaggcgcaggcgctggggcaggggcaggggctggggctggggcaggcgctggggcaggggctggggctggcgcaggcgcaggcgcaggggcaggcgcaggcgctggcgcaggcgccggggccggggccggcgctggcgcaggggctggcgctggggctggcgcaggcgctggcgctggggcaggggctggcgcaggcgctggggcaggcgcaggcgcaggggctggcgcaggggcaggcgcaggggctggggccggggccggggctggggcaggggccggggccggggctggcgctggggctggggctggcgcaggggctggggctggcgctggcgcaggggcaggcgctggggcaggggctggcgctggcgctggcgctggggctggggcaggcgcaggcgctggcgctggggctggcgctggcgctggggcaggggcaggcgctggcgccggggccggggccggggcaggggctggggcaggcgctggggctggggcaggggcaggcgcaggcgct

1. **GP**

atggcatacccatacgacgtcccagactacgccggacctggacctggcccaggacccggccctggaccaggacctgggcctggccctgggcctgggcccggaccaggacccggaccagggcctggacctggacccggacctggacctgggcctggccccggacctggccctggccctggaccaggacccggacccggcccaggacccggaccaggacccggccctggccctggccccggacccgggcctggacctggccctggacctggacccggccctgggcccggacctggaccaggccccggacctggacctggaccaggacccggaccaggacctgggcccggacctggccccggaccaggccccggacccggacctggccctggacccggacctgggcccggccccggccccggccccgggcccgggcccggccctggacctgggcctgggcctggacctggaccaggacctggccccggacccggacccggacctggacctggacccggccctggacctggcccaggacccggacctggccccggaccaggacctggacctgggcctgggcctggacccgggcctggacctggacccggaccaggaccaggacccggacctggacctggccctggacctgggcccggacctgggcctggccctggccctggccctggacctggccctggccccggacccggaccaggacccggacccggacccggaccaggacctggccctggaccaggacctggacccggacccggccctgggcct

1. **GR**

atgggcagaggaagaggcagaggcagaggcagaggaagaggaagaggcagaggcagaggcagaggaagaggaagaggaagaggcagaggaagaggaagaggaagaggcagaggcagaggaagaggaagaggcagaggcagaggaagaggaagaggcagaggcagaggaagaggaagaggcagaggcagaggaagaggaagaggcagaggcagaggaagaggaagaggcagaggcagaggaagaggaagaggcagaggcagaggaagaggaagaggcagaggcagaggaagaggaagaggcagaggcagaggaagaggaagaggcagaggcagaggaagaggaagaggcagaggcagaggaagaggaagaggcagaggcagaggaagaggaagaggcagaggcagaggaagaggaagaggcagaggcagaggaagaggaagaggcagaggcagaggaagaggaagaggcagaggcagaggaagaggaagaggcagaggcagaggaagaggaagaggcagaggcagaggaagaggaagaggcagaggcagaggaagaggaagaggcagaggcagaggaagaggaagaggcagaggcagaggaagaggaagaggcagaggcagaggaagaggaagaggcagaggcagaggaagaggaagaggcagaggcagaggaagaggaagaggcagaggcagaggaagaggaagaggcagaggcagaggaagaggaagaggcagaggcagaggaagaggcaga

1. **PA**

atgcctgctccagctcctgcaccagcaccagctccagctccagctcctgcaccagctccagcaccagcaccagcaccagctccagctccagctcctgctcctgctcctgctccagctcctgctcctgcaccagcaccagctccagcaccagcaccagcaccagctccagcaccagcaccagcaccagctccagctccagctccagcaccagctccagctccagctcctgctcctgctccagctcctgcaccagctccagctccagctcctgctccagcaccagcaccagctccagctccagctcctgctccagctccagctccagctcctgctcctgcaccagctccagctccagctccagcaccagcaccagctccagctccagctcctgctcctgctccagcaccagctccagctccagcaccagcaccagcaccagctccagctccagctcctgcaccagcaccagcaccagctccagctccagcaccagcaccagctccagctccagctcctgctcctgctcctgcaccagcaccagcaccagctccagctccagctcctgctccagcaccagctccagctccagctcctgctccagctccagctccagcaccagcaccagctccagctccagctcctgctccagctccagcaccagcaccagctccagctccagctcctgctcctgctccagctccagctcctgctcctgcaccagcaccagctccagctccagctcctgctcctgct

1. **PR**

atgcctagacctagaccaaggccaagacctaggccaagaccaaggcctagacctagacctagacctaggccaaggcctagacctagaccaaggcctagaccaagacctaggccaagacctagaccaaggccaaggccaagaccaagacctagaccaaggccaagacctagacctagaccaagaccaagaccaaggcctagacctaggccaaggccaaggccaagaccaaggccaaggcctagaccaagaccaagacctagacctagacctaggccaagaccaaggccaaggccaagacctagaccaagacctaggccaaggccaagaccaaggcctagacctagaccaagaccaagacctagaccaaggcctagaccaagaccaagacctaggccaagaccaagaccaagacctagaccaagaccaaggccaagacctaggccaaggcctagacctagacctagaccaaggccaaggcctagaccaaggccaagaccaagacctagacctagaccaaggcctagacctagacctaggccaagaccaagacctaggccaaggcctagacctaggccaagacctagaccaagaccaaggccaaggccaaggcctagacctaggccaagaccaagaccaagacctagacctagaccaagacctaggccaagacctagacctagacctagaccaagacctagacctaggccaaggcctagaccaagacctagaccaaggccaaggccaaggcctagg

**Figure 1S. DNA sequences of synthetic dipeptide repeats (DPR)**
